# Intravenous fluid prescribing errors in children: Mixed methods analysis of critical incidents

**DOI:** 10.1371/journal.pone.0186210

**Published:** 2017-10-12

**Authors:** Richard L. Conn, Steven McVea, Angela Carrington, Tim Dornan

**Affiliations:** 1 Centre for Medical Education, Queen’s University Belfast, Belfast, United Kingdom; 2 Neonatal Intensive Care Unit, Royal Jubilee Maternity Hospital, Belfast, United Kingdom; 3 Medicines Governance Team, Belfast Health and Social Care Trust, Belfast, United Kingdom; University Medical Center Goettingen, GERMANY

## Abstract

**Introduction:**

Recent National Institute for Health and Care Excellence (NICE) guidelines aim to improve intravenous (IV) fluid prescribing for children, but existing evidence about how and why fluid prescribing errors occur is limited. Studying this can lead to more effective implementation, through education and systems design.

**Aims:**

1. Identify types of IV fluid prescribing errors reported in practice
2. Analyse factors that contribute to errors
3. Provide guidance to educators and those responsible for designing systems

**Methods:**

Mixed methods observational study which analysed critical incident reports relating to IV fluid prescribing errors in children aged 0–16, occurring between 2011 and 2015 in UK secondary care. We quantified characteristics and types of errors, then qualitatively analysed narrative descriptions, identifying underlying contributing factors.

**Results:**

In the 40 incidents analysed, principal types of errors were incorrect rate of fluids, inappropriate choice of solution, and incorrect completion of prescription charts. Prescribers had to negotiate complex patients, interactions with other practitioners and teams, and challenging work environments; errors resulted from these inter-related contributing factors.

**Conclusions:**

This study highlights the diverse range and complex nature of IV fluid prescribing errors reported in practice. While these findings have the inherent limitations of critical incident reports, they point to areas of potential improvement in education and systems design. Practising prescribing in context, inducting doctors within the many specialties who contribute to care of children, and educating them in joint working with nurses and pharmacists could help reduce errors.

## Introduction

Intravenous (IV) fluid therapy is routine yet potentially lethal.[1] Whilst recent National Institute for Health and Care Excellence (NICE) guidelines[2] will help reduce risk, realising improvement needs a clearer understanding of error. Hyponatraemia has understandably dominated attention[3–14]; much less is known about other types of error or, importantly, their underlying causes. Finding out how and why IV fluid prescribing goes wrong could guide educators and help develop safer systems of care.

Prospective research involving children has been limited, and adult research is of limited applicability. Adult in-patients were affected by errors in calculating fluid rates, choosing types of fluid, and completing prescription charts.[15] Whilst these are self-evidently applicable to children, many specific aspects of prescribing differ, including methods of calculating rates of fluid administration, use of glucose-containing solutions, protocols, and charts. The little we know about paediatric prescribing comes mainly from small-scale audits, assessing particular types of errors. Errors in rate arose from miscalculation, use of incorrect formulae to calculate maintenance fluids,[6,11,14,16] and exceeding maximum allowed volumes.[7,17,18] Regarding fluid choice, even after 0.18% sodium chloride was withdrawn from use,[4,7,17] prescribers frequently prescribed hypotonic maintenance solutions (such as 0.45% sodium chloride),[18] even when hyponatraemia had developed.[4–7,9,13] They completed prescription charts incorrectly and omitted calculations and monitoring data.[4,13,14,17] Faced with this limited evidence base, NICE pragmatically recommended education to improve prescribers’ knowledge, and system changes such as standardising fluid prescription charts. But since imparting knowledge or introducing guidelines, alone, has little impact on doctors’ behaviour,[19] a more detailed analysis of the causes of errors could help target education more effectively, and advance NICE’s important work.

Our aim was to identify types of errors and explore contributing factors–*how* and *why* errors occur—to help make fluid therapy safer for children. Critical incidents–events reported by healthcare staff which cause actual or potential harm—have been established as a means to investigate errors. Reports provide categorical information, analysis of which enumerates the characteristics of errors, and narrative information, which helps identify causes. Researchers recently used this mixed methods approach to study patient safety issues and identify improvement opportunities in incident reports.[20,21] This article reports a mixed methods analysis of IV fluid prescribing incidents, categorising types of errors and identifying factors contributing to their occurrence.

## Methods

### Setting

This research was conducted within all five Health and Social Care Trusts in Northern Ireland, UK. Healthcare delivery in Northern Ireland is part of the National Health Service (NHS). Children are cared for in a broad range of settings: a large regional children’s hospital and neonatal unit; and several district general hospitals where inpatient paediatric wards provide medical and surgical care. Most hospitals also have maternity services and specialist neonatal units. Children may also receive care in non-paediatric settings, such as general emergency departments and specialist services that provide care for patients of all ages. In addition, children are typically moved to adult care once aged 14 or 15.

Staff voluntarily report critical incidents, using either an electronic database (Datix) available on hospital computers, or paper forms which are subsequently inputted to Datix. An example reporting form is available as S1 Form. All staff are encouraged to report incidents where harm occurred or there was a perceived risk of harm. Reports contain: patient demographics; where the incident occurred; what harm resulted; what type of incident it was; plus, a free text description of what happened and what subsequent action was taken.

Reports classified as medication incidents (including IV fluid incidents) are reviewed locally by medicines governance pharmacists (MGPs), whose role is similar to medication safety officers in other parts of the UK. They routinely review medication incidents and are trained in use of Datix. Categorical information within each reported incident is checked, including medication error type (prescribing/dispensing/administration/monitoring/other) and sub-type (wrong dose/wrong medicine etc.), drug(s) involved, and level of harm. Assignment of level of harm is guided by standardised risk matrices used in all Trusts (S1 Risk matrices).

### Data extraction

MGPs within each trust extracted from Datix all medication incidents in patients aged 0–16, occurring between July 2011 and July 2015. Recommended age limits for paediatric care and clinical guidelines made this age range a logical choice. Moreover, adolescents have experienced morbidity related to inappropriate IV fluid therapy,[22] reinforcing the argument that they should be treated similarly to other children. All reports from incidents occurring in children’s care settings were also extracted, to identify incidents where age had not been recorded.

Data was recorded in a pro forma in Microsoft Excel. We asked MGPs to follow a protocol detailing data extraction, processing and anonymisation. They rechecked categorical information and removed identifiable details, before transferring the fully anonymised dataset to the research team.

### Inclusion and exclusion criteria

RLC collated incidents involving IV fluid prescribing errors in a spreadsheet, double-checking the full dataset to ensure all were included. NICE guidelines were used to define IV fluids as ‘therapy to prevent or correct problems with fluid and/or electrolyte status’.[23] An attenuated dataset is available as S1 Dataset; an example incident with paraphrased narrative content is available as S1 Example incident.

To develop a full and authentic picture of IV fluid prescribing errors in practice, we included all incidents occurring across the five Trusts, including those where separate guidelines apply, such as neonatal care and diabetic ketoacidosis (DKA).

Two incidents were excluded: one involved a patient with hyponatraemia, which is a reporting trigger, but no error in IV fluid management was noted; the second involved heparinised saline used only to maintain central line patency, which fell outside the previously stated definition of IV fluids.

### Study design and analysis

Our mixed methods approach involved:

1. Reporting of incident characteristics with descriptive statistics
2. Identification, classification and quantification of error types from narrative descriptions
3. Thematic analysis of contributing factors, guided by Reason’s model of human error[24]

RLC initially quantified level of harm, age of patient affected, location of incident, and job role of reporter. Two authors (RLC and SMcV) then independently reviewed each incident, identifying types of error and developing a classification. A single incident could involve more than one error. Errors were defined as any reported deviation from accepted best practice at the time of study, with potential to cause harm. During the study period, practice in patients aged 0–16 was dictated by regional guidance, including a standardised chart,[25] and the 2007 NPSA safety brief.[26] Although NICE guidelines were not in place during the study period, and standards differed from recent recommendations, we felt there was value in contrasting types of error with current best practice. We therefore mapped error types to corresponding NICE recommendations.

The next stage was qualitative thematic analysis, employing Reason’s model of human error.[24] This ‘Swiss cheese model’ is commonly used for analysing prescribing errors,[27,28] working on the basis that harm occurs when deficiencies within a system align (Table 1).

**Table 1:** Incident analysis framework based on Reason’s model[24] (from Dornan et al[27], modified from Coombes et al[28]).

Condition	Example components
Latent conditions	• Organisational processes–workload, handwritten prescriptions • Management decisions–staffing levels, culture of lack of support for junior staff
Error-producing conditions	• Environmental–busy ward • Team–lack of supervision • Task–poor medication chart design • Patient–complex, communication difficulties
Active failures	• Slip, lapse, rule-based mistake, knowledge-based mistake
Defences	• Inadequate, unavailable, missing

Inter-rater reliability was addressed by two members of the research team (RLC and SMcV) independently coding data, using Reason’s four conditions as overarching categories. They resolved differences at all stages of analysis through discussion. By compiling and reviewing coded data relating to each aspect of Reason’s model, they jointly identified contributing factors underlying errors.

### Ethics

The Proportionate Review Sub-committee of the East Midlands—Nottingham 2 Research Ethics Committee—approved the research (reference 15/EM/0353). Governance approval was granted by each of the five Trusts.

## Results

### Characteristics

From a dataset of 517 prescribing incidents, 40 reports relating to IV fluids were included in the analysis. IV fluid prescribing incidents were third most commonly reported, after those involving antimicrobials and paracetamol. Characteristics are summarised in Table 2. Twelve incidents led to patient harm, of which all but one was insignificant or minor in severity. The incident graded as moderate involved hypoglycaemia, but did not mention lasting harm after initial treatment. No incidents involved severe harm. Half were deemed to have potential to cause moderate or severe harm had they not been intercepted. 38% of incidents occurred in children aged 12–16. Incidents occurred in a range of clinical areas, 32% of which were outside specific paediatric settings. 40% were reported by nursing staff.

**Table 2:** Characteristics of reported IV fluid incidents.

	Number of incidents (n = 40)	
Severity of harm	*Actual harm*	*Potential harm*
Insignificant	28 (70%)	2 (5%)
Minor	11 (27.5%)	18 (45%)
Moderate	1 (2.5%)	8 (20%)
Major	0 (0%)	12 (30%)
**Age of patient affected**		
0–27 days	6 (15%)	
28 days -12 months	1 (2.5%)	
13 months—2 years	3 (7.5%)	
2 years—5 years	9 (22.5%)	
6 years—11 years	3 (7.5%)	
12 years—16 years	15 (37.5%)	
Not specified	3 (7.5%)	
**Clinical area where incident occurred**		
Paediatric Medicine	14 (35%)	
Emergency Department	9 (22.5%)	
Surgery	6 (15%)	
Neonatal Unit	5 (12.5%)	
Adult medicine	1 (2.5%)	
Anaesthetics	1 (2.5%)	
Gynaecology	1 (2.5%)	
Unknown	3 (2.5%)	
**Who reported the incident**		
Medical staff	9 (22.5%)	
Nursing staff	16 (40%)	
Unknown	15 (37.5%)	

### Types of errors

There were 70 errors in the 40 incidents, the principal types of which were: incorrect rate (27); inappropriate choice (17); and incorrect completion of fluid prescription chart (16) (Table 3).

**Table 3:** Types of IV fluid prescribing errors reported.

Code	Category	Corresponding NICE recommendation	Number (n = 70)
1	**Incorrect rate**		**27 (39%)**
**1.1**	Exceeding the maximum rate of maintenance fluids	1.4.1^a^	12 (17%)
**1.2**	Incorrect calculation of rate	1.4.1 ^b^	11 (16%)
**1.3**	Issues with patient weight	1.2.1	2 (3%)
**1.4**	Other	-	2 (3%)
2	**Inappropriate choice**		**17 (24%)**
**2.1**	Inappropriate concentration of glucose in fluids	^c^	6 (9%)
**2.2**	Inappropriate choice of electrolyte content in fluid	1.4.6	5 (7%)
**2.3**	Failure to use an appropriate maintenance fluid	1.4.3^d^	3 (4%)
**2.4**	Failure to use an isotonic crystalloid in resuscitation	1.3.1	1 (1%)
**2.5**	Other	-	2 (3%)
3	**Incorrect completion of fluid prescription chart**		**16 (23%)**
**3.1**	Omission of information	1.2.3	7 (10%)
**3.2**	Inappropriate use of adult fluid prescription chart	^e^	6 (9%)
**3.3**	Other	-	3 (4%)
4	**Other errors**		**10 (14%)**
**4.1**	Failure to use individualised protocols in specific situations	1.3.4	6 (9%)
**4.2**	Failure to adjust fluids to reflect clinical change	1.2.3	4 (6%)

^a^ NICE suggest that ‘males rarely need more than 2500 ml and females rarely need more than 2000 ml of fluid’. We identified errors within this category according to a similar local policy which limits rate of IV fluids to 100ml/hour in males and 80ml/hour in females.

^b^ Prescribing of fluid rate involved proper application of the appropriate formula e.g. Holliday-Segar formula in calculating maintenance fluids.

^c^ NICE recognise that there is a lack of evidence regarding the appropriateness of glucose in IV fluids in children. Errors involved: not prescribing a glucose containing fluid despite a specific indication, such as metabolic disease, hypoglycaemia with vomiting, or DKA; and a 10% glucose containing fluid being prescribed inappropriately to an 11 year old.

^d^ The three instances identified involved: 5% glucose; 0.45% sodium chloride in an operative patient; 0.45% sodium chloride in a hyponatraemic patient.

^e^ We identified errors when the regional chart for patients aged 0–16 was not used. While use of a specific paediatric chart is not mandated by NICE, adoption of standardised charts is an area in which research is recommended.

### Analysis of contributing factors

**Fig 1:**
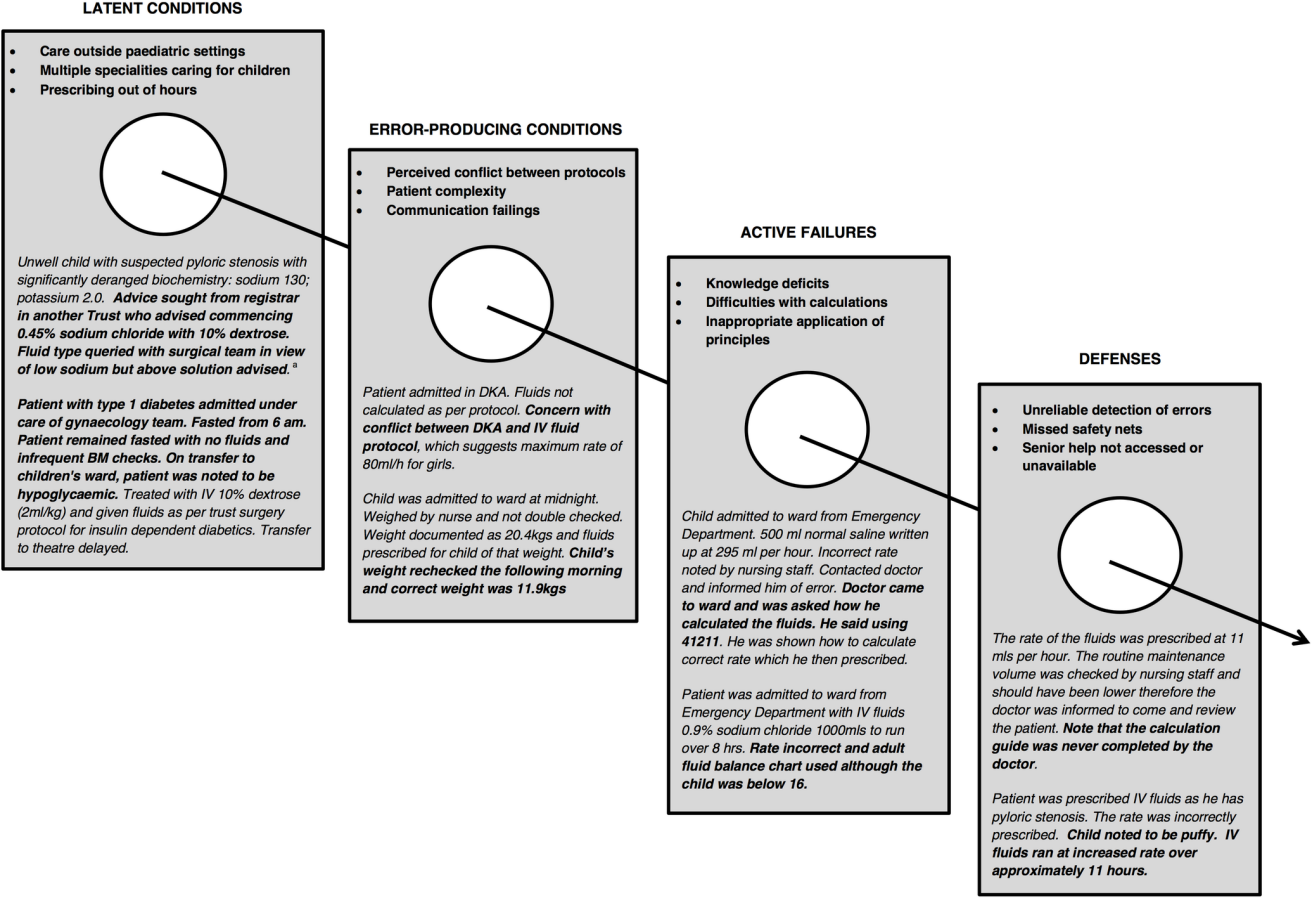
Factors contributing to IV fluid errors. Examples are paraphrased; bold added by authors for emphasis.

Contributing factors are summarised in Fig 1.

#### Latent conditions

Organisational factors—non-specialist care settings (that is, those in which staff are not specifically trained in the care of children as, for example, paediatricians or paediatric nurses are), and doctors in a range of specialties prescribing for children—contributed to errors. Several involved failure to use paediatric charts and exceeding advised maximum rates of fluids, particularly in children looked after on adult wards. Errors arose when approaches used in adult practice were applied to children, exemplified by an adolescent being prescribed a litre of 5% dextrose over four hours. Reporters frequently noted prescribing errors occurring outside normal working hours. In some of these cases, not accessing support from a senior, supervising doctor, or an appropriate sub-specialist, was a factor.

#### Error-producing conditions

Features of the patient, task, environment, or team were involved in most errors. Chronic illnesses, such as diabetes, renal disease, and metabolic disease, made management more complex. In these cases, prescribers often had difficulty negotiating additional protocols, whether patient-specific, as in metabolic conditions, or disease-specific, as in DKA. In some cases, there was perceived conflict between protocols: for example, a reporter noted that following a DKA protocol meant exceeding the advised maximum rate of fluids. Patients with rapidly changing clinical conditions were error-prone, such as when glucose was not included in the initial IV fluid prescription for a child with vomiting, leading to hypoglycaemia. A ‘task’ feature identified was prescribers frequently omitting body weights or rate calculations from charts. Sometimes this was because the requisite paediatric charts were not available.

Poor communication within teams contributed to errors. One incident describes a prescription being amended to correct hypokalaemia, but not being administered as the change was not communicated. In another, nursing staff gave fluids based on a verbal instruction which was later found to be different from the written prescription. Occasionally, incidents resulted from disagreement between staff. For example, an anaesthetist persistently requested that fluids be administered at an excessive rate despite a nurse’s concerns that this was incorrect.

#### Active failures

Active failures occurred across the range of types described by Reason. Knowledge-based mistakes were seen in miscalculation of IV fluid rates. Often these resulted from unfamiliarity with the Holliday-Segar formula,[29] or difficulty applying it. More common were rule-based mistakes, in which doctors understood principles of prescribing fluids for children, but did not apply these appropriately in specific contexts. For example, a child with a urea cycle disorder was treated with IV fluids which, while usually appropriate, contained too much sodium and insufficient glucose. There were also examples of ‘unintended actions’ causing errors–slips, like prescribing fluids on the wrong patient’s chart, and lapses, such as forgetting to monitor. Occasionally, violations occurred, such as choosing to use an adult fluid balance chart—often to save time—or knowingly exceeding the maximum recommended rate of fluids.

#### Defences

‘Defences’ refers to systems or staff actions to detect errors, prevent them reaching patients, or mitigate their effects. Nine errors were intercepted before fluids were given; most others were recognised before any significant harm occurred. By their nature, reported incidents relate to errors which have been picked up, and not those that go undetected. It follows that incidents contain information about successful defences, as well as missed opportunities to detect errors earlier.

Five errors were detected during mandatory pre-administration checks; 24 errors were missed during checking and detected after fluids had been started. The checking process appeared unreliable and depended heavily on vigilance of individual staff. Most errors were noted only when patients moved between wards (16 instances); their care was taken over by new staff (four); or their clinical condition changed (four).

Sometimes staff did not use intended safeguards, such as when they used adult rather than paediatric charts or failed to complete calculation guides incorporated within charts. In another case, a doctor and nurse bypassed pre-administration checks and administered IV fluids prior to the prescription being written. Some incidents indicated vulnerabilities in systems: one involved a patient being inaccurately weighed without any procedure for double checking; in another, the existence of two seemingly discrepant protocols made it difficult to identify unsafe practice.

The ability of staff to act as a defence depended not just on their vigilance but their level of expertise; some errors in complex patients went undetected until sub-specialist teams (e.g. paediatric metabolic team) reviewed them. Prescribers sometimes delayed seeking help from senior doctors or involving sub-specialists.

## Discussion

### Summary and context

Like adults in previous studies,[15] children were endangered by incorrect rates, inappropriate choices, and incorrect charting of fluids. Many specific types of error occurred, most unrelated to hyponatraemia or use of hypotonic fluids. Most errors seen relate to aspects of practice deemed key priorities in recent NICE guidelines.[2]

Interactions between individual, social, organisational, and environmental factors contributed to errors; knowledge deficits tended to be contextual rather than factual. To practise safely, prescribers had to negotiate challenging workplaces, navigate protocols, communicate effectively, use multiple resources, and correctly apply knowledge and rules. These findings resonate with those from earlier authors who showed prescribing to be a complex and inherently contextual process.[27,28,30] As reported before,[16] factors leading to errors were more likely to affect prescribers who were more junior, and less familiar with treating children.

Clinical audit and Northern Ireland paediatric admissions data[31] suggest that tens of thousands of intravenous fluid prescriptions were written during the period when the 40 errors occurred. This suggests that intravenous fluids are generally safe, but underlines the importance of the topic, given how frequently they are prescribed.

### Strengths and limitations

A strength of the study was that it used a large, pre-existing critical incident dataset to gain insights into authentic clinical practice and drive improvements. We maintained rigour throughout. We adopted a comprehensive strategy to capture data. Experienced medicines governance pharmacists vetted incidents, both after initial reporting, and following data extraction. This made it more likely that all incidents were captured, and that categorical components such as level of harm were accurate. Two authors independently coded the dataset at the data analysis stage. Discussion of findings encouraged reflexivity and careful consideration of data limitations during interpretation. We only reported themes about which we reached consensus, and have presented examples of narrative data in support of these. All members of the research team, chosen to represent a range of disciplines, agreed on the final analysis.

We recognise the limitations of using critical incidents. Firstly, these are subject to under-reporting.[32] This may partly explain why only 40 incidents were reported in a time period when thousands of prescriptions were written. Reporting is also selective and motivations to report differ between staff groups.[33,34] The fact that, in our study, more incidents were reported by nurses than doctors, may reflect this. Reporting biases could have affected the type of incidents seen, and the underlying contributing factors identified.

Secondly, incident reports can be incomplete and of variable quality. Some fields had data missing, such as who had reported the incident. Other potentially useful information, such as the grade of doctor responsible, was not routinely reported. Furthermore, the way incident reports were written made it difficult to get information about some aspects of Reason’s model; for example, whether a doctor made a knowledge-based or rule-based mistake. Similarly, staff did not usually comment on contextual factors that led to errors, such as distractions or clinical pressures.

These factors limit the conclusions that can be drawn, particularly, from our quantitative data. Whilst these cannot be considered representative, they are nevertheless informative. Existing evidence (as described previously) is limited. Most comes from clinical audits focussing on limited aspects of fluid prescribing, such as use of hypotonic fluids. Given this limited evidence base, educators and quality improvers can use our findings about the many different types of errors seen to improve practice, pending more representative data from prospective research.

Qualitative analysis differs in that insights are drawn from the words within narrative descriptions, not how frequently events occur. By their nature, critical incidents contain information pertinent to patient safety. We used the rich evidence within these accounts to elicit factors contributing to errors. We were guided by the strength of the evidence presented, and its importance to safe fluid prescribing. In this way, the validity of our conclusions does not depend on representativeness. Our findings are transferable to other settings and contribute to research priorities identified by NICE.

### Recommendations and conclusion

Our research recommendation is for prospective studies to advance the epidemiology of errors. Table 4 summarises educational recommendations. Undergraduate paediatric placements should teach fluid prescribing. The induction of all doctors who treat children, not just paediatricians, should teach how to prescribe fluids and make best use of information resources and clinical guidelines. Given the contextual nature of errors, learners need to practise prescribing, under supervision, and in context, before prescribing ‘solo’. Specific training should address special clinical situations, such as DKA or neonatal care. Interprofessional education, finally, could promote safe, collaborative practice and help nurses intercept errors.

**Table 4:** Recommendations for IV fluid prescribing education.

Ensure IV fluid prescribing is included in undergraduate paediatric placements, including opportunities to practice the skill in-situ under supervision
Deliver specific postgraduate induction for all groups of doctors expected to prescribe for children
Consider opportunities for interprofessional education, bringing together doctors, nurses and pharmacists
Provide specific training in managing special scenarios eg. DKA and using resources eg. paediatric fluid prescription charts

Our clinical recommendations (Table 5) are that prescribing could be made safer in pressured clinical services by not treating children in adult wards,[35] using paediatric fluid balance charts with built-in prescribing safeguards, and ensuring clinical pharmacists are available and involved.[36] It is hoped this will replicate the positive impact they have had on prescribing other drugs.[37,38] Newer solutions such as electronic prescribing could offer opportunities to improve safety, by making safeguards more difficult to bypass. Critical incidents can also draw attention to specific systems improvements; for example, errors resulting from incorrectly recording patient weight could be prevented by a double checking system. Our study demonstrates the potential benefit from large-scale analysis of critical incidents; we recommend that, as well as being used locally, IV fluid prescribing incidents be collated, studied and shared more broadly.

**Table 5:** Recommendations for systems changes to improve IV fluid prescribing.

Avoid treating children in adult wards
Use specific paediatric fluid balance charts with built-in safeguards such as calculation guides and guidance about fluid choice
Ensure adequate provision of paediatric clinical pharmacists and involve them in reviewing IV fluid prescriptions
Consider solutions such as electronic prescribing, with built-in safeguards which cannot be easily bypassed
Use critical incident data, ideally on a regional level, to identify potential areas for improvement

Given the complex nature of the problem, it is unlikely any single measure will be fully effective. Complex interventions, incorporating some or all of the above measures, are most likely to succeed in making fluid prescribing safer.

## Supporting information

S1 Form Example incident reporting form. (PDF)

S1 Risk matrices Risk matrices used within Trusts to assign incidents level of actual and potential severity. (PDF)

S1 Dataset Attenuated version of study dataset. Narrative content has been removed to protect confidentiality of patients and staff involved.
(XLSX)

S1 Example incident Example of a critical incident, including paraphrased narrative information. (PDF)
