# Supplementary material for: Intravenous fluid prescribing errors in children: Mixed methods analysis of critical incidents
Source: PLoS One. 2017 Oct 12;12(10):e0186210. doi: 10.1371/journal.pone.0186210 (PMC5638410; doi:10.1371/journal.pone.0186210)
Supplement: S1 Form — (PDF) [file pone.0186210.s001.pdf]

BHSCT Incident Report Form

Click for: General Datixweb Completion Tips • Approving Managers Guidance Pack • Password Reset Instructions • Changes following UPGRADE (16.04.15)

1. Approving Manager - If name is not on the list - DO NOT COMPLETE FORM - and seek advice from line manager as only areas with a trained approver can use datixweb to report incidents

* Approving manager's name

2. Incident Details

* What happened?

- Enter facts, not opinions.
- Ensure that CAPS LOCK is OFF.
- DO NOT use peoples names
- DO NOT use peoples initials
- Use job titles or patient/client
- Use third person i.e. DON'T use: I, my, we etc

* Immediate action taken (including action to prevent re-occurrence).

Enter action taken at the time of the incident

Who has been notified of this incident?

* Incident date (dd/MM/yyyy)

Time: 24hr clock format i.e. (hh:mm)

3. Where did the incident happen?

* Site

* Location (type)

* Location (exact)

* Directorate

* Service Area

* Specialty

4. Incident Coding

* Incident Type

* Result

* Severity (ACTUAL)

Click for BHSCT Severity / Impact Table.

5. Person(s) Affected or Harmed section

If there is more than one person was affected, use the "ADD ANOTHER" button at the end of section 5 to add the additional person(s) details. **Clear Section**

* Person Affected/Victim: Person Affected / Victim

* Type

* Contact Sub Type

First Name(s)

Surname

Multiple Contact ID (e.g. patient A)

Gender

Date of birth (dd/MM/yyyy)

Address

Postcode

Date of death (dd/MM/yyyy)

* Was the person injured?

Add Another

6. Other Person(s) Involved Section

* Was any other person(s) involved?

7. Equipment Details

* Was any equipment, medical device or accessory involved in the incident?

8. Documents

* Do you have document(s) you wish to attach to this record?

Clear Section

* Contact type

* Contact Sub Type

* First Name(s)

* Surname

Your Work Telephone Number:

Your Staff Number:

Your Email (*@belfasttrust.hscni.net*)

Submit | Submit and print | Cancel

DatixWeb 12.3.6.3 © Datix Ltd 2015
