# Supplementary material for: Intravenous fluid prescribing errors in children: Mixed methods analysis of critical incidents
Source: PLoS One. 2017 Oct 12;12(10):e0186210. doi: 10.1371/journal.pone.0186210 (PMC5638410; doi:10.1371/journal.pone.0186210)
Supplement: S1 Risk matrices — (PDF) [file pone.0186210.s002.pdf]

S1 Risk matrices. Risk matrices used within Trusts to assign incidents level of actual and potential severity.

DOMAIN	IMPACT (CONSEQUENCE) LEVELS [can be used for both actual and potential]				
	**INSIGNIFICANT (1)**	**MINOR (2)**	**MODERATE (3)**	**MAJOR (4)**	**CATASTROPHIC (5)**
**PEOPLE**<br>*(Impact on the Health/Safety/Welfare of any person affected: e.g. Patient/Service User, Staff, Visitor, Contractor)*	• Near miss, no injury or harm.	• Short-term injury/minor harm requiring first aid/medical treatment.<br>• Any patient safety incident that required extra observation or minor treatment e.g. first aid<br>• Non-permanent harm lasting less than one month<br>• Admission to hospital for observation or extended stay (1-4 days duration)<br>• Emotional distress (recovery expected within days or weeks).	• Semi-permanent harm/disability (physical/emotional injuries/trauma) (Recovery expected within one year).<br>• Admission/readmission to hospital or extended length of hospital stay/care provision (5-14 days).<br>• Any patient safety incident that resulted in a moderate increase in treatment e.g. surgery required	• Long-term permanent harm/disability (physical/emotional injuries/trauma).<br>• Increase in length of hospital stay/care provision by >14 days.	• Permanent harm/disability (physical/ emotional trauma) to more than one person.<br>• Incident leading to death.
**QUALITY & PROFESSIONAL STANDARDS/ GUIDELINES**<br>*(Meeting quality/ professional standards/ statutory functions/ responsibilities and Audit Inspections)*	• Minor non-compliance with internal standards, professional standards, policy or protocol.<br>• Audit / Inspection – small number of recommendations which focus on minor quality improvements issues.	• Single failure to meet internal professional standard or follow protocol.<br>• Audit/Inspection – recommendations can be addressed by low level management action.	• Repeated failure to meet internal professional standards or follow protocols.<br>• Audit / Inspection – challenging recommendations that can be addressed by action plan.	• Repeated failure to meet regional/ national standards.<br>• Repeated failure to meet professional standards or failure to meet statutory functions/ responsibilities.<br>• Audit / Inspection – Critical Report.	• Gross failure to meet external/national standards.<br>• Gross failure to meet professional standards or statutory functions/ responsibilities.<br>• Audit / Inspection – Severely Critical Report.
**REPUTATION**<br>*(Adverse publicity, enquiries from public representatives/media Legal/Statutory Requirements)*	• Local public/political concern.<br>• Local press < 1day coverage.<br>• Informal contact / Potential intervention by Enforcing Authority (e.g. HSENI/NIFRS).	• Local public/political concern.<br>• Extended local press < 7 day coverage with minor effect on public confidence.<br>• Advisory letter from enforcing authority/increased inspection by regulatory authority.	• Regional public/political concern.<br>• Regional/National press < 3 days coverage. Significant effect on public confidence.<br>• Improvement notice/failure to comply notice.	• MLA concern (Questions in Assembly).<br>• Regional / National Media interest >3 days < 7days. Public confidence in the organisation undermined.<br>• Criminal Prosecution.<br>• Prohibition Notice.<br>• Executive Officer dismissed.<br>• External Investigation or Independent Review (eg, Ombudsman).<br>• Major Public Enquiry.	• Full Public Enquiry/Critical PAC Hearing.<br>• Regional and National adverse media publicity > 7 days.<br>• Criminal prosecution – Corporate Manslaughter Act.<br>• Executive Officer fined or imprisoned.<br>• Judicial Review/Public Enquiry.
**FINANCE, INFORMATION & ASSETS**<br>*(Protect assets of the organisation and avoid loss)*	• Commissioning costs (£) <1m.<br>• Loss of assets due to damage to premises/property.<br>• Loss – £1K to £10K.<br>• Minor loss of non-personal information.	• Commissioning costs (£) 1m – 2m.<br>• Loss of assets due to minor damage to premises/ property.<br>• Loss – £10K to £100K.<br>• Loss of information.<br>• Impact to service immediately containable, medium financial loss	• Commissioning costs (£) 2m – 5m.<br>• Loss of assets due to moderate damage to premises/ property.<br>• Loss – £100K to £250K.<br>• Loss of or unauthorised access to sensitive / business critical information<br>• Impact on service contained with assistance, high financial loss	• Commissioning costs (£) 5m – 10m.<br>• Loss of assets due to major damage to premises/property.<br>• Loss – £250K to £2m.<br>• Loss of or corruption of sensitive / business critical information.<br>• Loss of ability to provide services, major financial loss	• Commissioning costs (£) > 10m.<br>• Loss of assets due to severe organisation wide damage to property/premises.<br>• Loss – > £2m.<br>• Permanent loss of or corruption of sensitive/business critical information.<br>• Collapse of service, huge financial loss
**RESOURCES**<br>*(Service and Business interruption, problems with service provision, including staffing (number and competence), premises and equipment)*	• Loss/ interruption < 8 hour resulting in insignificant damage or loss/impact on service.<br>• No impact on public health social care.<br>• Insignificant unmet need.<br>• Minimal disruption to routine activities of staff and organisation.	• Loss/interruption or access to systems denied 8 – 24 hours resulting in minor damage or loss/ impact on service.<br>• Short term impact on public health social care.<br>• Minor unmet need.<br>• Minor impact on staff, service delivery and organisation, rapidly absorbed.	• Loss/ interruption 1-7 days resulting in moderate damage or loss/impact on service.<br>• Moderate impact on public health and social care.<br>• Moderate unmet need.<br>• Moderate impact on staff, service delivery and organisation absorbed with significant level of intervention.<br>• Access to systems denied and incident expected to last more than 1 day.	• Loss/ interruption 8-31 days resulting in major damage or loss/impact on service.<br>• Major impact on public health and social care.<br>• Major unmet need.<br>• Major impact on staff, service delivery and organisation - absorbed with some formal intervention with other organisations.	• Loss/ interruption >31 days resulting in catastrophic damage or loss/impact on service.<br>• Catastrophic impact on public health and social care.<br>• Catastrophic unmet need.<br>• Catastrophic impact on staff, service delivery and organisation - absorbed with significant formal intervention with other organisations.
**ENVIRONMENTAL**<br>*(Air, Land, Water, Waste management)*	• Nuisance release.	• On site release contained by organisation.	• Moderate on site release contained by organisation.<br>• Moderate off site release contained by organisation.	• Major release affecting minimal off-site area requiring external assistance (fire brigade, radiation, protection service etc).	• Toxic release affecting off-site with detrimental effect requiring outside assistance.

HSC REGIONAL RISK MATRIX – WITH EFFECT FROM APRIL 2013 (updated June 2016)

Risk Likelihood Scoring Table			
**Likelihood Scoring Descriptors**	**Score**	**Frequency**<br>**(How often might it/does it happen?)**	**Time framed Descriptions of Frequency**
Almost certain	5	Will undoubtedly happen/recur on a frequent basis	Expected to occur at least daily
Likely	4	Will probably happen/recur, but it is not a persisting issue/circumstances	Expected to occur at least weekly
Possible	3	Might happen or recur occasionally	Expected to occur at least monthly
Unlikely	2	Do not expect it to happen/recur but it may do so	Expected to occur at least annually
Rare	1	This will probably never happen/recur	Not expected to occur for years

	**Impact (Consequence) Levels**				
**Likelihood Scoring Descriptors**	**Insignificant(1)**	**Minor (2)**	**Moderate (3)**	**Major (4)**	**Catastrophic (5)**
**Almost Certain (5)**	**Medium**	**Medium**	**High**	**Extreme**	**Extreme**
**Likely (4)**	**Low**	**Medium**	**Medium**	**High**	**Extreme**
**Possible (3)**	**Low**	**Low**	**Medium**	**High**	**Extreme**
**Unlikely (2)**	**Low**	**Low**	**Medium**	**High**	**High**
**Rare (1)**	**Low**	**Low**	**Medium**	**High**	**High**

Severity rating of medication related incidents

Immediate assessment should be performed quickly, even when all facts may not be available. There is always scope to re-grade as facts and issues emerge.

Actual impact on patient (factual)

Outcome of the actual incident in terms of harm

Severity of incident	Definition<br>Any medication related event resulting in:
Catastrophic	Death
Major	Near death<br>Permanent harm
Moderate	Treatment being given<br>Temporary significant harm<br>Prolonged patient stay
Minor	Minor harm<br>Increased patient monitoring
Insignificant	No harm

Potential future risks to patients in your organisation
Consider the same facts reoccurring but without any factors which prevented the actual impact on the patient being more severe

Most likely consequences of medicine related incident if it were to occur again (if in doubt grade up, not down)

Likelihood of recurrence	Insignificant<br>No harm	Minor<br>Minor harm<br>Increased patient monitoring	Moderate<br>Treatment<br>Temporary significant harm<br>Prolonged patient stay	Major<br>Near death<br>Permanent harm	Catastrophic<br>Death
Almost certain					
Likely					
Possible					
Unlikely					
Rare					

Risk rating

	Very low	
	Low	
	Moderate	
	High	
