# Supplementary material for: Intravenous fluid prescribing errors in children: Mixed methods analysis of critical incidents
Source: PLoS One. 2017 Oct 12;12(10):e0186210. doi: 10.1371/journal.pone.0186210 (PMC5638410; doi:10.1371/journal.pone.0186210)
Supplement: S1 Example incident — (PDF) [file pone.0186210.s004.pdf]

S1 Example incident. Example of a critical incident, including paraphrased narrative information.

Trust	Site	Location[a]	Reference number	Age at time of incident[a]	Description of incident[b,c]	Action taken[b]	Reporter (job role)[a]	Drug involved	Incident type	Datix CCS code	Outcome (actual severity)[a]	Consequence (potential severity)[a]
Trust A	Site A	Emergency Department	-	2 years - 5 years	4 year old boy attended emergency department, with vomiting and moderate dehydration. Blood sugar was initially 3.8 [mmol/L]. Treated with intravenous and oral fluids. His blood sugar continued to drop. It was subsequently noted that the child had been prescribed maintenance [IV] fluids without dextrose.	Correct fluids prescribed and administered. Child admitted for treatment of dehydration. All of above discussed with parents. The following morning, incident was discussed with [senior nurse].	Consultant/ Professor	IV fluids	Medication error during the prescription process	Wrong/ unclear drug / medicine	Insignificant	Minor

[a] Categorical data used in reporting characteristics (Table 2)
[b] Narrative data used to identify types of errors (Table 3)
[c] Narrative data used in qualitiative analysis to elicit contributing factors to errors (Fig1)
